# Changes in use of breast-conserving therapy in years 1978-2000.

**DOI:** 10.1038/bjc.1994.466

**Published:** 1994-12

**Authors:** H. J. de Koning, J. A. van Dongen, P. J. van der Maas

**Affiliations:** Department of Public Health, Erasmus Universiteit, Rotterdam, The Netherlands.

## Abstract

The treatment of breast cancer patients has changed rapidly in the past decade, but empirical data at local and national level are scarce. Predicting the consequences of screening for primary treatment is consequently difficult. The aim of this analysis of records on admissions to hospital of women with breast cancer and/or for breast surgery (1975-90) together with a survey of all Dutch radiotherapy departments (1986-88) is to show the change in breast-conserving therapy and other primary treatment before the start of breast cancer screening in The Netherlands. There was a modest increase in breast-conserving therapy after 1981, coinciding with the first publication on its trial, followed by a sharp increase between 1985 and 1990, after the second publication. At the end of that 5 year period, 36% of all women with newly diagnosed invasive breast cancer underwent this type of surgery. Breast-conserving surgery is always followed by radiotherapy, but there has been a clear reduction in post-operative radiation after mastectomy. The percentage of breast-conserving therapy is at present higher in The Netherlands than in the USA. Implementing the Dutch screening programme will result in a maximum increase in breast-conserving therapy at national level of 34%, which stabilises at +21%, or a 50% maximum increase at local level. The number of women treated by mastectomy will ultimately decrease by 9%. Given the rapidity of change towards the use of breast-conserving surgery, which is enhanced by screening, recent information will be needed in predicting capacity and assessing whether screen-detected women are treated adequately.


Br. J. Cancer (1994), 70, 1165–1170 © Macmillan Press Ltd., 1994

### Changes in use of breast-conserving therapy in years 1978–2000

H.J. de Koning[1], J.A. van Dongen[2] & P.J. van der Maas[1]

[1]*Department of Public Health, Erasmus Universiteit, PO Box 1738, 3000 DR Rotterdam, The Netherlands;* [2]*Antoni van Leeuwenhoekhuis/Netherlands Cancer Institute, Department of Surgery, Plesmanlaan 121, 1066 CX Amsterdam, The Netherlands.*

**Summary** The treatment of breast cancer patients has changed rapidly in the past decade, but empirical data at local and national level are scarce. Predicting the consequences of screening for primary treatment is consequently difficult. The aim of this analysis of records on admissions to hospital of women with breast cancer and/or for breast surgery (1975–90) together with a survey of all Dutch radiotherapy departments (1986–88) is to show the change in breast-conserving therapy and other primary treatment before the start of breast cancer screening in The Netherlands. There was a modest increase in breast-conserving therapy after 1981, coinciding with the first publication on its trial, followed by a sharp increase between 1985 and 1990, after the second publication. At the end of that 5 year period, 36% of all women with newly diagnosed invasive breast cancer underwent this type of surgery. Breast-conserving surgery is always followed by radiotherapy, but there has been a clear reduction in post-operative radiation after mastectomy. The percentage of breast-conserving therapy is at present higher in The Netherlands than in the USA. Implementing the Dutch screening programme will result in a maximum increase in breast-conserving therapy at national level of 34%, which stabilises at + 21%, or a 50% maximum increase at local level. The number of women treated by mastectomy will ultimately decrease by 9%. Given the rapidity of change towards the use of breast-conserving surgery, which is enhanced by screening, recent information will be needed in predicting capacity and assessing whether screen-detected women are treated adequately.

The treatment of breast cancer patients has changed rapidly in the past decade. Several trials have led to a better understanding of the possible results of different types of primary treatment. Breast-conserving therapy, limited surgery followed by high-dose radiation, has been shown to be as effective as modified radical mastectomy for most operable patients (Veronesi *et al.*, 1981). For mastectomy patients, routine post-operative radiotherapy is now usually considered unnecessary (Edland, 1988; Harris & Hellman, 1988). It could be assumed that treatment practice would be influenced by the information from these trials, but empirical data at national and local level are scarce in most countries (Farrow *et al.*, 1992; Chouillet *et al.*, 1994).

At the same time, countries have started to implement breast cancer screening, as trials have shown that a reduction in mortality from breast cancer can be expected for screened women aged 50–69. The impact on the number and type of surgical procedures and radiotherapy will be great as a result of both the temporary increase in the number of women detected with breast cancer and the increase in early cancers (de Koning *et al.*, 1990). Bottlenecks in capacity are expected, but there is a lack of empirical data to support this hypothesis.

As we were especially uncertain about the development of breast-conserving therapy (outside a screening programme), we have analysed data on the actual number and type of primary treatments for breast cancer in The Netherlands in the years before the start of nationwide screening. In this report, we present the trend in primary treatment for the period 1975–90, and the expected influence of breast cancer screening in the near future. Emphasis is placed on surgery and radiotherapy, and especially on the comparison between breast-conserving therapy and mastectomy, and on post-operative radiotherapy after mastectomy. The findings are compared with the scarce international data, from the USA, and may serve for planning and evaluation of both breast cancer treatment and screening, and for the interpretation of the impact of results from new trials.

### Materials and methods

Two independent sources provided data on the use of surgery and/or radiotherapy for women with breast cancer. The number of different types of surgical procedures was determined by analysing all the available records on hospital admissions in The Netherlands for women with breast cancer and/or for breast surgery for the period 1975–90 (Centre for Health Care Information). Coverage of recording increased in this period from 83% of all admissions (in 1975) to, respectively, 89%, 90%, 94%, 94%, 95%, 94% (in 1981), 97%, 98%, 99% (in 1984–89) to 99.7% in 1990. The records included the patient's age, whether it was a first or subsequent admission, a detailed description of surgical procedures, number of nursing days, diagnoses at discharge and residence after discharge. The accuracy of recording surgical procedures can be assumed to be high, since its registration is based on a uniform and detailed classification of surgery codes (Classification of Surgery, 1977) with 25 different types of breast surgery. It is being applied in all hospitals by trained personnel with national guidelines for recording breast-conserving therapy. Only in 1990, a revised (and extended) coding system was introduced.

Data on radiation treatment are not recorded centrally in The Netherlands. A questionnaire was therefore sent to all 20 Dutch radiotherapy departments, concerning the number of female breast cancer patients who had had radiotherapy and the different types of radiotherapy in the years 1986–88. Sixteen departments responded, and additional information on the total patient population of all 20 departments enabled us to extrapolate figures for the whole population.

Except for one regional cancer register, no incidence data were available from a complete national record of cancer. The clinical (age-specific) breast cancer incidence was based on the national registration of first hospital admissions and on an additional 15% of breast cancers in the 70 + group, assumed to be underrepresented in incidence figures based on hospital admissions (e.g. for primary hormonal treatment). National data that have recently become available for the year 1990 confirm this (NCR, 1994).

All primary breast cancer treatment could be divided into treatment for women with ductal carcinoma *in situ* (DCIS) (treatment by either local excision, local excision plus radiotherapy or total mastectomy) and that for women with invasive carcinoma. The latter group is treated by breast-conserving therapy with an external booster, breast-conserving therapy with an iridium implant, total mastectomy, total mastectomy with post-operative radiotherapy (all usually with axillary dissection), a combination of treatment modalities for stage IIIB tumours or primary hormonal treatment (tamoxifen); see also Table I. For these treatment

Correspondence: H.J. de Koning.
Received 4 January 1994; and in revised form 9 June 1994.

**Table I** Estimated numbers and percentages of new breast cancer treatments in The Netherlands in 1990 without mass screening

| *Treatment* | *Numbers* | *%* | *Main sources* |
|---|---|---|---|
| Primary treatment DCIS | | | |
| Local excision | 115 | 30 | Hospital; trial |
| Local excision + radiotherapy | 115 | 30 | Hospital; trial |
| Total mastectomy | 150 | 40 | Hospital |
| Total | 380 | 100 | |
| Primary treatment invasive carcinoma | | | |
| Breast-conserving therapy, external booster | 2,400 | 30 | Hospital; radiotherapy |
| Breast-conserving therapy, iridium implant | 475 | 6 | Hospital; radiotherapy |
| Mastectomy, no radiotherapy | 2,400 | 30 | Hospital; radiotherapy |
| Matectomy and radiotherapy | 1,525 | 19 | Hospital; radiotherapy |
| Primary tamoxifen | 375 | 5 | Model |
| Treatment locally advanced disease (IIIB) | 400 | 5 | Registry |
| Stage IV treatment | 450 | 55 | Registry |
| Total | 8,025 | 100 | |
| Total female breast cancer incidence | 8,405[a] | | |
| Adjuvant tamoxifen | 1,750 | 26[b] | Survey |
| Adjuvant chemotherapy | 750 | 11[b] | Survey |
| Total | 2,500 | 37 | |

[a]In 1994, data from the national cancer register on the 1990 newly registered cancer cases became available (NCR, 1994); adjusting for the 355 screen-detected cases, national incidence is approximately 8,575, 2% higher than previously estimated. [b]Percentage related to invasive carcinomas, except stages IIIB and IV and already primary tamoxifen.

options, and for adjuvant systemic treatment, we estimated the number of women treated in The Netherlands in 1990. The year 1990 was chosen for practical reasons, since it coincided with the start of nationwide screening.

Combining these figures with the clinical incidence and stage distribution of breast cancer, and the treatment guidelines, we predicted the chance of being treated by each modality for four groups of women: those with clinically diagnosed ductal carcinoma *in situ*; those with invasive carcinomas smaller than 10 mm in diameter; those with carcinomas larger than or equal to 10 mm but smaller than 20 mm; and those with invasive carcinomas of 20 mm and more. The axillary lymph node status corresponding with these stages was taken into account. With screening, there is a shift towards smaller sizes and towards a more favourable lymph node status. Women with screen-detected cancer have a lower percentage of axillary lymph node metastases than women with clinically diagnosed cancer, even within the same tumour size category (de Koning *et al.*, 1990; Tabár *et al.*, 1992). Lymph node and distant metastases status for screen-detected cancers was determined from the Utrecht and Nijmegen experimental screening projects, and for clinically diagnosed cancers from the Utrecht 'non-screened' group. The chances of treatment modality per tumour size category were also predicted for women with screen-detected breast cancer (see Appendix).

The MISCAN breast cancer model (van Oortmarssen *et al.*, 1990*a*) was used to predict the number of women in the four tumour categories with and without breast cancer screening. The disease model is based on a three-state division of the development of invasive carcinoma and one DCIS state. Key parameters on the mean duration of the screen-detectable preclinical states and the sensitivity were derived from results of the Health Insurance Plan (HIP) analysis (van Oortmarssen *et al.*, 1990*b*) and from a new analysis of all results from the Dutch screening trials in Nijmegen and Utrecht (de Koning *et al.*, 1991). This method has been shown to be useful for predicting the effects and cost of screening in other countries, e.g. Australia and Germany, using country-specific data if available (Carter *et al.*, 1993; Beemsterboer *et al.*, 1994) or using data from other screening projects (Boer *et al.*, 1994). In this report, the influence of screening on the change in treatments will be shown for a policy of 2-yearly screening for women aged 50–69 and an attendance rate of 70%, which started in 1990 and will be fully implemented around 1994. The projected changes in management in the years when screening is gradually being introduced might be influenced by so-called autonomous changes, such as a continuous rise in the use of breast-conserving therapy independent of the detection of more smaller lesions. Additionally, a survey among 40 Dutch breast cancer experts was used to estimate the possible trend in primary treatment in the 1990s if there were no screening programme. Three variants were distinguished in the forecasts: one in which the number of women treated by breast-conserving therapy will continue to follow the forecast increase to 1995. In this scenario, we assume – from the data analysed – that this technology is further spread during these years. In a second variant the proportion will remain at the 1990 level (treatment criteria are stable) and will only be influenced by the increase in breast cancer incidence. A third, intermediate variant was also distinguished.

### Results

### *Primary surgery and/or radiotherapy 1978–90*

The main treatment choice for women with operable invasive breast cancer is between conservation or more radical treatment. In the period 1978–80, the number of recorded admissions for limited breast surgery for breast cancer was very small, and probably not all related to breast-conserving therapy (Figure 1a). Even in the preceding 3 years there were as many as 135 such admissions recorded annually, although this treatment was not really standard practice in those years, as far as we know. From 1981, coinciding with the publication of the first results of the randomised trial on quadrantectomy, there was a relatively small absolute increase in breast-conserving therapy. However, a rapid and steady increase from 1985 on is visible, the year the first results of the second randomised trial were published. Ultimately, 2,823 hospital admissions for limited breast cancer surgery were recorded in 1990 (including approximately 200 screen-detected cases).

The independent survey among radiotherapy departments confirms the rapid and steady increase in the number of radiation treatments as part of breast-conserving therapy for

**Figure 1** **a**, Number of admissions for women with breast cancer and limited breast surgery, 1978–90, The Netherlands: 94.3% coverage in 1978, 94.0% in 1981, 98.9% in 1985, 99.7% in 1990. **b**, Annual number of breast cancer patients treated by (high-dose) radiation after limited breast surgery (breast-conserving therapy), as registered in each radiotherapy department (+), as a percentage of the total annual number of all patients radiated (not only breast cancer patients). Δ Mean per cent of all departments, The Netherlands 1986–88. On average, total (*n*) 23,350. Total numbers in 1987 or 1988 differ only 1% from those in 1986, and may therefore be used as a steady baseline for the individual department and years. The decrease in post-operative radiotherapy after mastectomy would complicate comparisons if the percentage was related to all breast patients.

the years 1986–88. Figure 1b shows the annual number of breast cancer patients treated by (high-dose) radiation after limited breast surgery (as a percentage of the total number of all patients with radiotherapy) in the different departments. Although there is variation between centres, the increase in the average total number equals that seen in records of hospital admissions (see also Figure 2). In these years 1,330, 1,679 and 2,015 treatments respectively were recorded in 16 departments. If these centres are assumed to be a representative sample of all 20 Dutch centres, the estimate for the whole of The Netherlands would be 2,400 treated women in 1988. About 15% of these treatments are followed by an iridium booster, the others by an external booster.

The differences in numbers between the two sources are relatively small (Figure 2). Although there has been no linkage between the two data sources, given the 99% coverage of hospital records and the similar trend when analysing the radiotherapy data, we can conclude that both sources on surgery and radiotherapy are in agreement with each other. The hospital records probably slightly underestimate the breast-conserving therapy as each year another 200 admissions are coded as 'biopsy and axillary dissection' (not included in the above estimates), whereas the radiotherapy data might slightly overestimate its numbers owing to a less uniform recording and no distinction between invasive and non-invasive cancer. We therefore used the average estimate for the present situation (Figure 2, line C), that approximately 2,900 women had been treated conservatively in 1990, which is 36% of all women with newly diagnosed invasive breast carcinoma in The Netherlands.

**Figure 2** Annual numbers of patients with invasive breast cancer treated by limited surgery followed by radiotherapy (breast-conserving therapy) in The Netherlands, 1983–2000. (A) according to national hospitals' records; (B) according to radiotherapy departments; (C) without screening, 1990 level (no trend); (D) with screening (base level C); (E) without screening, 1992 level (including time trend); (F) with screening (base level E) (G) maximum number with screening (including time trend and expert opinion). *Number of patients treated by radiation after mastectomy (radiation departments).

Except for a small proportion of women treated by primary hormonal treatment or by primary radiotherapy, the remaining women are treated with breast ablation, mostly by modified radical mastectomy. Whether this should be followed by radiotherapy on a routine basis is a matter of dispute. Fifteen radiotherapy departments recorded a clear decrease in the number of women treated by radiation after a mastectomy, from 2,126 in 1986 to 1,518 in 1988 (Figure 2, line*). The decrease in 1987–88 is less evident than in the previous 2 years, and it remains difficult to establish a definite trend. Nevertheless, on the basis of these data, we assume a total of 1,525 post-mastectomy irradiations in 1990, which corresponds to 39% of the breast cancer patients treated by mastectomy. Table I summarises the estimated numbers of treatment modalities applied in 1990.

### *Possible developments in 1990–2000 independent of mass screening*

Further developments are to be expected in the near future on at least the criteria for performing breast-conserving therapy and for radiotherapy after mastectomy, thereby possibly resulting in autonomous changes independent of screening. Experts envisage an increase in the proportion of breast cancer patients treated by breast-conserving therapy in the next 5–10 years, from the present 35% up to 50% of all female breast cancer patients, if there were no screening programme. This would mean continuation of the line seen in 1985–90. They foresee room for a further spreading of the technology throughout the country as a result of more and better multidisciplinary treatment protocols, change of treatment criteria, greater demand from women and/or a generally earlier diagnosis of breast cancer. This expected increase will inevitably level off, as there will come a time when all patients who are eligible for conservative surgery and want it are actually treated in this fashion. In practice, the upper limit of tumour size tends to decrease owing to unsatisfactory cosmetic results in larger tumours. When factors such as the presence of extensive ductal carcinoma *in situ* around the invasive part, the refusal of women or doctors and the tumour size/breast volume ratio are taken into account, we can assume that 50% of all women with operable invasive breast cancer (but excluding stage IIIb, not stage IV, not primary tamoxifen) might be treated conservatively. This represents approximately 42% of all newly diagnosed invasive breast cancer patients per year, which would thus have been reached around 1992 (medium variant, Figure 2, line E).

**Figure 3** Annual change (%) in breast-conserving therapy (**a**) and breast ablation (**b**) at local and national level resulting from the screening of women aged 50–69 every 2 years (percentage compared with situation without mass screening in that year). National level (■): gradual build-up of all screening units over 5 years. Regional level ( ■ ): consequences surrounding one screening unit with approximately 10,000 screens per year starting in 1990 (2 year build-up).

*Influence of mass screening in 1990–2000*

The introduction of mass screening has a profound impact on these estimates. Implementation will always result in a temporary increase in the number of persons detected who have the disease. Most evident is the increase in detected cancers at the first (prevalent) screening. The start of the Dutch breast cancer screening programme in 1990 resulted in a rise in the number of newly diagnosed cases of 355. A maximum 17% (= 1,450 cancers) increase is expected in the year 1993. From 1996 onwards, the total yearly number of diagnosed breast cancers will be 3.5% higher than the number without screening (de Koning *et al.*, 1991).

The main consequences of earlier diagnosis for treatment practice are shown in Figure 3, taking 1990 as a stable situation. Since almost all treatments depend on at least tumour type and size and/or lymph node metastases, we may expect differences to occur for all therapeutic aspects. Most important is the shift from mastectomy to breast-conserving therapy owing to the detection of cancer at an earlier stage. At a national level, we would expect a steady increase in breast-conserving therapy up to a 34% increase (+ 1,025) in 1994. After that, the increase will stabilise at approximately + 21% (+ 640 per year) compared with the number of treatments expected without screening each year (Figure 2, line D, and Figure 3a). The number of women treated by mastectomy will ultimately decrease by 9% (– 370 per year). The decrease in the number of mastectomies does not equal the increase in breast-conserving therapy because of the higher incidence and detection of early lesions resulting from screening. All primary treatments that will eventually show a decrease owing to the more favourable stage distribution of screen-detected cancers still show an increase in the build-up period of screening. This is a result of both the temporary increase in women diagnosed as having cancer and the less favourable stage distribution in which these cancers are detected at the first screening round compared with subsequent rounds.

At the regional level, the temporary increase in the number of breast-conserving therapies may be much more dramatic, since the regional build-up periods of screening are even shorter than the nationwide build-up. Figures 3a and b also show the percentage changes for the region surrounding a centre with 10–12,000 screens a year that started in 1990: the number of women who undergo breast-conserving therapy increases by 50% to begin with, but does not last as long.

### Discussion

*Increase in breast-conserving therapy*

The number of breast cancer patients treated conservatively has increased rapidly in The Netherlands since the mid-1980s, coinciding with the appearance of results of the second trial (Fisher *et al.*, 1985). It is clear that earlier estimates based on 1983–85 admission data only are outdated as a result of this change (de Koning *et al.*, 1990). Given the high coverage of (detailed) recording, and the similarity in data from two independent sources, it is highly unlikely that the observed trend has been influenced by any recording biases. However, the increase is not infinite, as trials have shown that breast-conserving therapy is only as effective as modified radical mastectomy for localised small cancers, and several studies have shown definitive contraindications (Bartelink *et al.*, 1988; van Dongen *et al.*, 1992). Data from The Netherlands Cancer Institute suggest that approximately 55% of all operable patients are appropriate candidates for breast-conserving therapy (Hooning *et al.*, 1991). The present analysis shows two important aspects in terms of the quality of breast cancer treatment in The Netherlands: breast-conserving surgery is always followed by radiation treatment and the actual number of women treated conservatively is rapidly approaching the number that would be expected on the basis of oncological data and protocols. The extent to which breast-conserving therapy has been adopted in other countries is rarely described. Only recently, information became available from parts of the USA (Farrow *et al.*, 1992). In nine areas, the percentage of white women ($n = 18,399$ in 4 years) with localised invasive breast cancer treated by breast-conserving surgery increased from approximately 22% in the period 1983–84 to 33% in 1985–86, which seems to be an extremely early increase in this technology. It is known that the publication in 1985 of the results of the US randomised clinical trial of breast-conserving surgery led to a temporary peak in some districts in 1985, followed by a decline (Lazovich *et al.*, 1991). A publication on a much larger group of 41,680 breast cancer patients treated in nine other areas in the USA in 1988 – 31% of all cases in that year – reveals only 25% of these patients being treated by partial mastectomy (Osteen *et al.*, 1992). It is apparent that there are strong regional differences in the USA, and that the often heard proposition that Europe follows developments in the USA after a time lag does not seem to be borne out by the data on breast-conserving therapy in The Netherlands. The percentage has been higher in the latter country in recent years. Secondly, although there was a nationwide temporal trend towards the increasing use of radiation after breast-conserving surgery in the USA, the situation there was quite different from the situation in The Netherlands, where all women received radiotherapy after limited surgery.

*Future treatment changes*

The uncertainties are more or less concentrated in the upper ranges of our results. Of course, future practice in radiation treatment after mastectomy would seem to be the biggest unknown factor. It might be said that we are unable to predict these numbers in a situation in which screening is implemented in the next few years. Almost all experts thought it very likely that the percentage of women that would undergo radiation treatment after mastectomy would decrease in the next 5–10 years (without taking account of a screening programme). It is likely that even less radiation treatment will be given after radical mastectomy, but that it will continue to decrease both for women with screen-detected cancers and for those with clinically diagnosed cancers. The influence of new treatment trials or new results of ongoing trials have, of course, not yet been taken into account (Veronesi *et al.*, 1993). Most of these apply to treatment schedules in which a less serious intervention is compared with the present treatments described in our results.

Even the influence of mass screening on breast-conserving therapy alters if we assume that the results of treatment trials had not yet influenced some clinicians' and/or women's decisions. If we assume that the increase in breast conservation will continue until 1995, mainly as a result of a general broadening of criteria, we might expect that both women with breast cancer diagnosed clinically and women with screen-detected breast cancer would be treated more frequently by conservation in the nearby future (Figure 2, line G). An important advantage of this analysis is the prediction of treatment changes before a screening programme has started, which can be used to estimate the required capacity. The initial increases in breast-conserving surgery, mastectomy and DCIS treatment as a result of screening may be relatively small in terms of the total number of surgical procedures, but represent more than 7.5% of present breast surgery. Together with the additional increase in biopsies, the early years of screening will result in an additional average increase in breast surgery at national level of 15–20%. Even more important would be to forecast changes for radiotherapy services, as investment in possible new machinery takes time. Considering both the increase in primary radiation treatment due to screening and the decreases in treatment for advanced disease (de Koning *et al.*, 1992), the total number of radiotherapeutic sessions would increase by 5% in 1994, which is 22% of all sessions for breast cancer. This increase will initially have to be dealt with using existing facilities.

*Expectations compared with first observations*

There is almost no literature on the predicted treatment consequences of implementing screening. This seems a logical result of the lack of national data on applied treatment modalities for breast cancer in most countries where screening is not carried out, and of the lack of a method in which the detailed changes induced by screening, in terms of number, stage and period of detected cancers, are taken into account (as in this study). The first published results on changes in breast-conserving therapy due to screening support our analysis at the local level. Although numbers are small, in the Enschede region breast-conserving therapy increased by 50–70% compared with the situation without screening (Boekema *et al.*, 1992). Sixty-one per cent of screen-detected (invasive) breast cancer patients underwent conservation surgery, compared with 43% of patients diagnosed outside the screening. Recent data from another large region, the IKA region, show that approximately 40% of operable (invasive) screen-detected patients underwent conservative surgery (1990–92), but there is wide variation between hospitals (17–71%) (P.C.M. van Velthoven, personal communication, 1993). But, again, the difference induced by screening is striking, as in 1989 only approximately 30% of the breast cancer patients were treated conservatively (without screening). The projections of screening in general, as we have made for The Netherlands, appear to resemble closely the actual performance of screening in the first years; the attendance rate and the number and stages of cancers detected for the 180,000 newly invited women compare favourably with the expected values (NETB, 1994).

We will be following the actual changes in the next years, at both national and regional levels. Important regional differences should be monitored when screening is implemented and, together with the stage distribution, these findings may function as a guide as to whether screened women with breast cancer are treated adequately. Detailed information on treatment changes in respect of mass screening are also crucial for estimating the influence on the quality of life, and for adjusting the expected future reduction in breast cancer mortality, if necessary. In particular, the increasing use of adjuvant systemic treatment and consequent mortality reduction may interact with the achievable reduction arising from earlier detection of cancer.

In conclusion, the rapidity of change towards the use of breast-conserving surgery, like the change from radical mastectomy to modified radical mastectomy during the 1970s, will be enhanced by the additional increase due to mass screening.

We would especially like to thank the Dutch Society for Radiotherapy for its voluntary contribution to the study, and in particular Professor Dr G.M.M. Bartelink and Professor J.W.H. Leer. Our thanks go to all the radiotherapy departments and persons that completed and returned the questionnaires. Our thanks also go to Dr P.C.M. van Velthoven, medical auditor, IKA region, for supplying regional data. We would like to acknowledge the contribution made by Mrs A.E. de Bruyn and Mrs P.M.M. Beemsterboer.

### References

BARTELINK, H., BORGER, J.H., VAN DONGEN, J.A. & PETERSE, J.L. (1988). The impact of tumor size and histology on local control after breast-conserving therapy. *Radiother. Oncol.*, **11**, 297–303.

BEEMSTERBOER, P.M.M., DE KONING, H.J., WARMERDAM, P.G., BOER, R., SWART, E., DIERKS, M.-L. & ROBRA, B.-P. (1994). Prediction of the effects and costs of breast-cancer screening in Germany. *Int. J. Cancer*, **58**, 623–628.

BOEKEMA, A.G., DORNSEIFFEN, G., MULDER, H.J., VOS, R.A.I. & KLUFT-DE HAAS, B.A. (1992). The first experience with breast cancer screening in Enschede region. I. Effectiveness (in Dutch). *Ned. Tijdschr. Geneeskd.*, **136**, 1757–1760.

BOER, R., WARMERDAM, P., DE KONING, H. & VAN OORTMARSSEN, G. (1994). Extra incidence caused by mammographic screening (letter). *Lancet*, **343**, 979.

CARTER, R., GLASZIOU, P., VAN OORTMARSSEN, G.J., DE KONING, H.J., STEVENSON, C., SALKELD, G. & BOER, R. (1993). Cost-effectiveness of mammographic screening in Australia. *Austr. J. Publ. Hlth.*, **17**, 42–50.

CHOUILLET, A.M., BELL, C.M.J. & HISCOX, J.G. (1994). Management of breast cancer in southeast England. *Br. Med. J.*, **308**, 168–171.

VAN DONGEN, J.A., BARTELINK, H., FENTIMAN, I.S., LERUT, T., MIGNOLET, F., OLTHUIS, G., VAN DER SCHUEREN, E., SYLVESTER, R., TONG, D., WINTER, J. & VAN ZIJL, K. (1992). Factors influencing local relapse and survival and results of salvage treatment after breast-conserving therapy in operable breast cancer: EORTC Trial 10801, breast conservation compared with mastectomy in TNM stage I and II breast cancer. *Eur. J. Cancer*, **28A**, 801–805.

EDLAND, R.W. (1988). Presidential address: does adjuvant radiotherapy have a role in the postmastectomy management of patients with operable breast cancer – revisited. *Int. J. Radiat. Oncol. Biol. Phys.*, **15**, 519–535.

FARROW, D.C., HUNT, W.F. & SAMET, J.M. (1992). Geographic variation in the treatment of localized breast cancer. *N. Engl. J. Med.*, **326**, 1097–1101.

FISHER, B., BAUER, M., MARGOLESE, R., POISSON, R., PILCH, Y., REDMOND, C., FISHER, E., WOLMARK, N., DEUTSCH, M., MONTAGUE, E., SAFFER, E., WICKERHAM, L., LERNER, H., GLASS, A., SHIBATA, H., DECKERS, L., KETCHAM, A., OISHI, R. & RUSSELL, I. (1985). Five-year results of a randomized clinical trial comparing total mastectomy and segmental mastectomy with or without radiation in the treatment of breast cancer. *N. Engl. J. Med.*, **312**, 665-673.

HARRIS, J.R. & HELLMAN, S. (1988). Put the 'hockey stick' on ice. *Int. J. Radiat. Oncol. Biol. Phys.*, **15**, 497-499.

HOONING, M.J., VAN DONGEN, J.A. & WENT, G. (1991). Changing indications for breast conserving therapy: proportion of patients with operable breast cancer suitable for breast conservation. *Neth. J. Surg.*, **43**, 102-104.

DE KONING, H.J., VAN OORTMARSSEN, G.J., VAN INEVELD, B.M. & VAN DER MAAS P.J. (1990). Breast cancer screening: its impact on clinical medicine. *Br. J. Cancer*, **61**, 292-297.

DE KONING, H.J., VAN INEVELD, B.M., VAN OORTMARSSEN, G.J., DE HAES, J.C.J.M., COLLETTE, H.J.A., HENDRIKS, J.H.C.L. & VAN DER MAAS, P.J. (1991). Breast cancer screening and cost-effectiveness; policy alternatives, quality of life considerations and the possible impact of uncertain factors. *Int. J. Cancer*, **49**, 531-537.

DE KONING, H.J., VAN INEVELD, B.M., DE HAES, J.C.J.M., VAN OORTMARSSEN, G.J., KLIJN, J.G.M. & VAN DER MAAS, P.J. (1992). Advanced breast cancer and its prevention by screening. *Br. J. Cancer*, **65**, 950-955.

LAZOVICH, D., WHITE, E., THOMAS, D.B. & MOE, R.E. (1991). Underutilization of breast-conserving surgery and radiation therapy among women with stage I or II breast cancer. *J. Am. Med. Assoc.*, **266**, 3433-3438.

NATIONAL EVALUATION TEAM FOR BREAST CANCER SCREENING (1994). Nationwide breast cancer screening in The Netherlands and breast cancer mortality reduction (poster). Lancet conference *The Challenge of Breast Cancer*, Brugge, 1994.

NCR (NETHERLANDS CANCER REGISTRY) (1994). *Incidence of Cancer in The Netherlands 1990*, a report. NCR: Utrecht.

VAN OORTMARSSEN, G.J., HABBEMA, J.D.F., VAN DER MAAS, P.J., DE KONING, H.J., COLLETTE, H.J.A., VERBEEK, A.L.M., GEERTS, A.T. & LUBBE, J.TH.N. (1990*a*). A model for breast cancer screening. *Cancer*, **66**, 1601-1612.

VAN OORTMARSSEN, G.J., HABBEMA, J.D.F., LUBBE, J.TH.N. & VAN DER MAAS, P.J. (1990*b*). A model-based analysis of the HIP project for breast cancer screening. *Int. J. Cancer*, **46**, 207-213.

OSTEEN, R.T., STEELE, G.D., MENCK, H.R. & WINCHESTER, D.P. (1992). Regional differences in surgical management of breast cancer. *CA - Cancer J. Clin.*, **42**, 39-43.

TABÁR, L., FAGERBERG, G., DUFFY, S.W., DAY, N.E., GAD, A. & GRÖNTOFT, O. (1992). Update of the Swedish two-county program of mammographic screening for breast cancer. *Radiol. Clin. N. Am.*, **30**, 187-210.

VERONESI, U., SACCOZZI, R., DEL VECCHIO, M., BANFI, A., CLEMENTE, C., DE LENA, M., GALLUS, G., GRECO, M., LUINI, A., MARUBINI, E., MUSCOLINO, G., RILKE, F., SALVADORI, B., ZECCHINI, A. & ZUCALI, R. (1981). Comparing radical mastectomy with quadrantectomy, axillary dissection, and radiotherapy in patients with small cancer of the breast. *N. Engl. J. Med.*, **305**, 6-11.

VERONESI, U., LUINI, A., DEL VECCHIO, M., GRECO, M., GALIMBERTI, V., MERSON, M., RILKE, F., SACCHINI, V., SACCOZZI, R., SAVIO, T., ZUCALI, R., ZURRIDA, S. & SALVADORI, B. (1993). Radiotherapy after breast-preserving surgery in women with localized cancer of the breast. *N. Engl. J. Med.*, **328**, 1587-1591.

### Appendix

(a) Chance of breast-conserving therapy for operable women with invasive breast cancer (not stage IIIb, not stage IV, not primary tamoxifen): 1990 level (no trend)

| | *Size of tumour (mm)* | | |
|---|---|---|---|
| | *<10* | *10-19* | *≥20* |
| Clinically diagnosed | 0.7 | 0.6 | 0.3 |
| Screen-detected | 0.8 | 0.7 | 0.5 |

(b) Chance of post-operative radiotherapy for operable women with invasive carcinoma treated by mastectomy: 1990 level (no trend)

| | *Size of tumour (mm)* | | |
|---|---|---|---|
| | *<10* | *10-19* | *≥20* |
| Clinically diagnosed | 0.30 | 0.33 | 0.41 |
| Screen-detected | 0.25 | 0.29 | 0.35 |


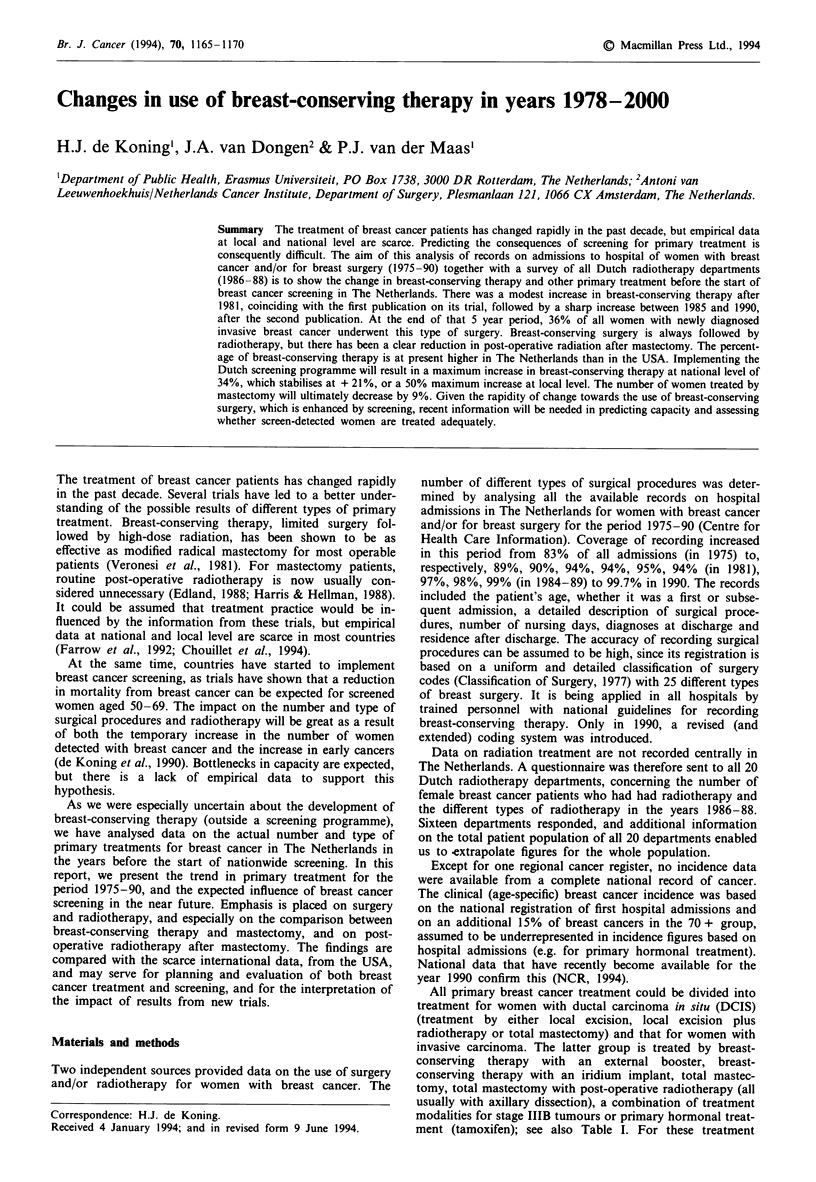

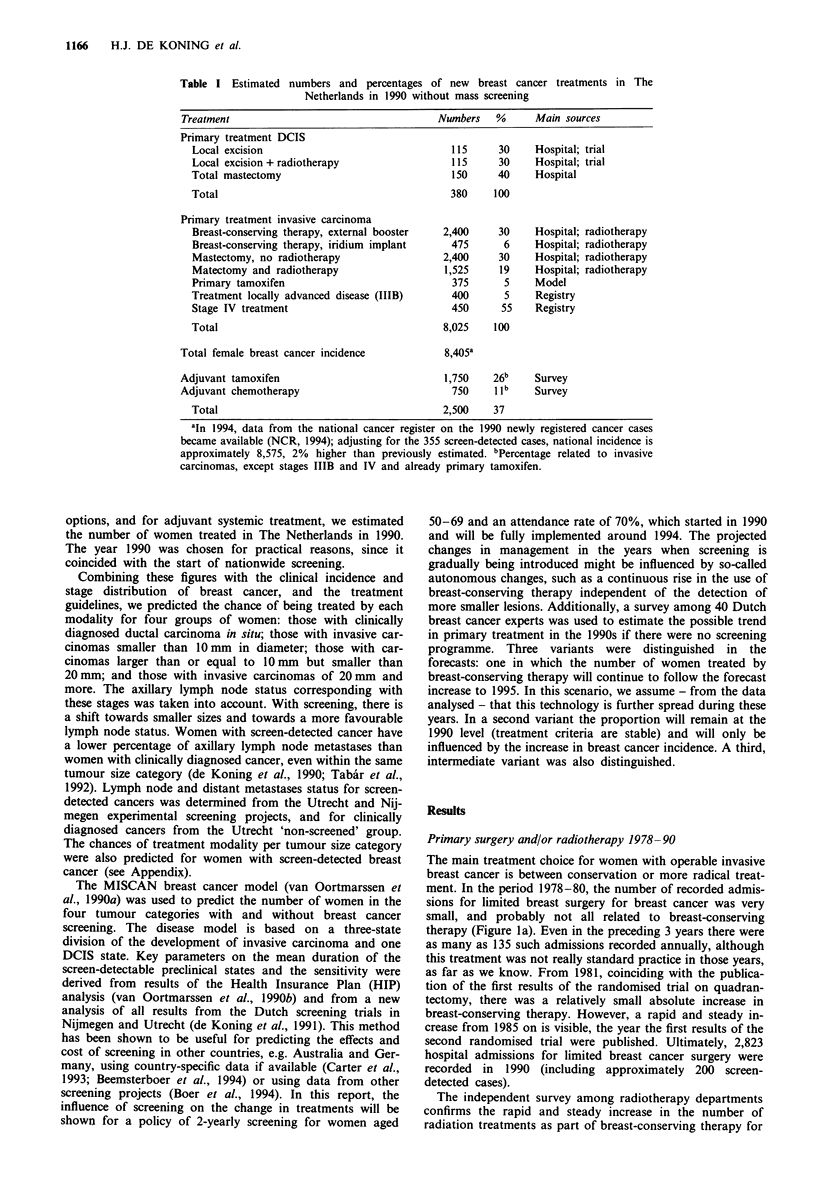

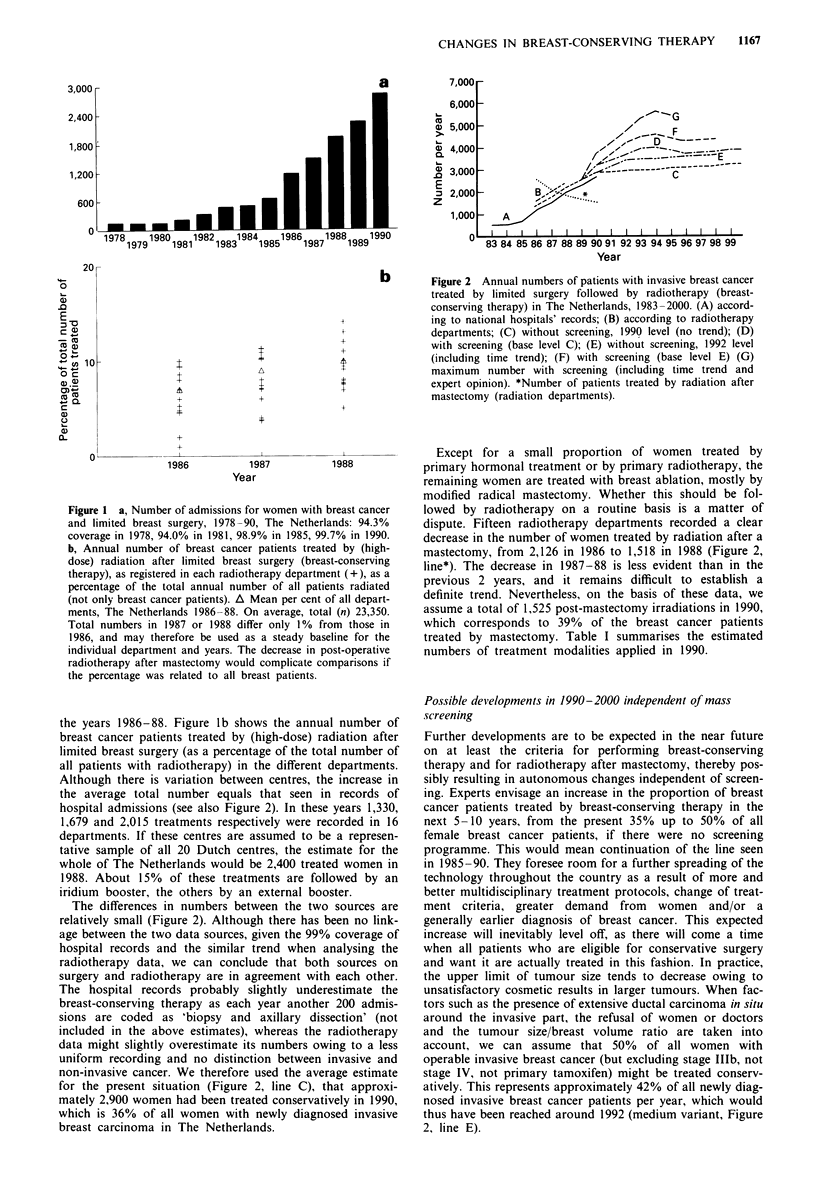

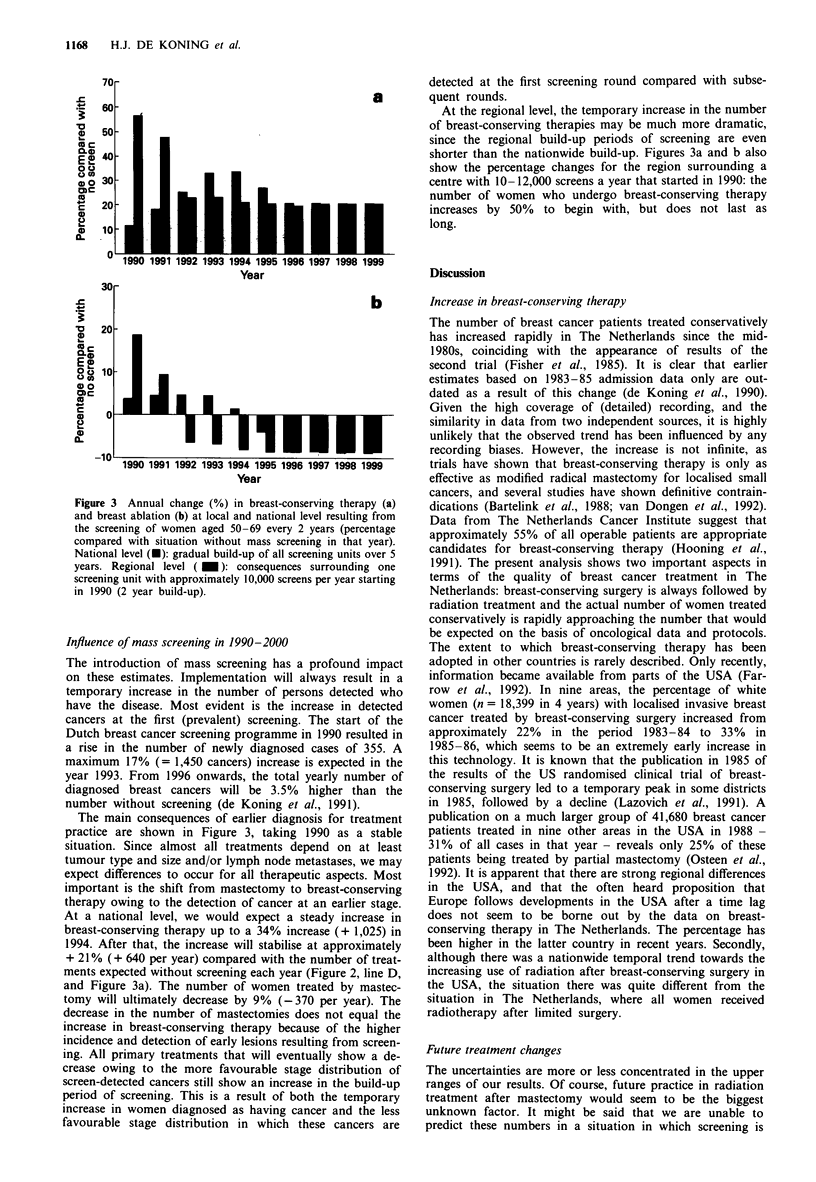

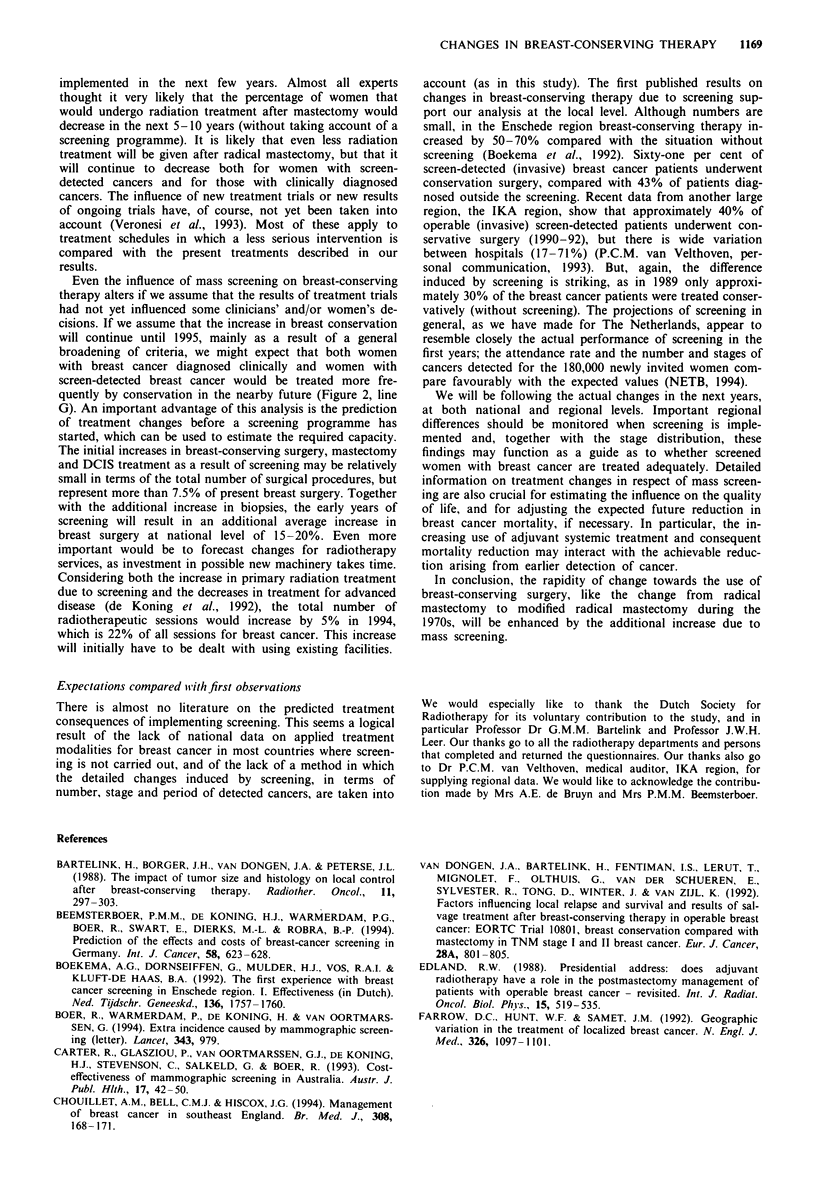

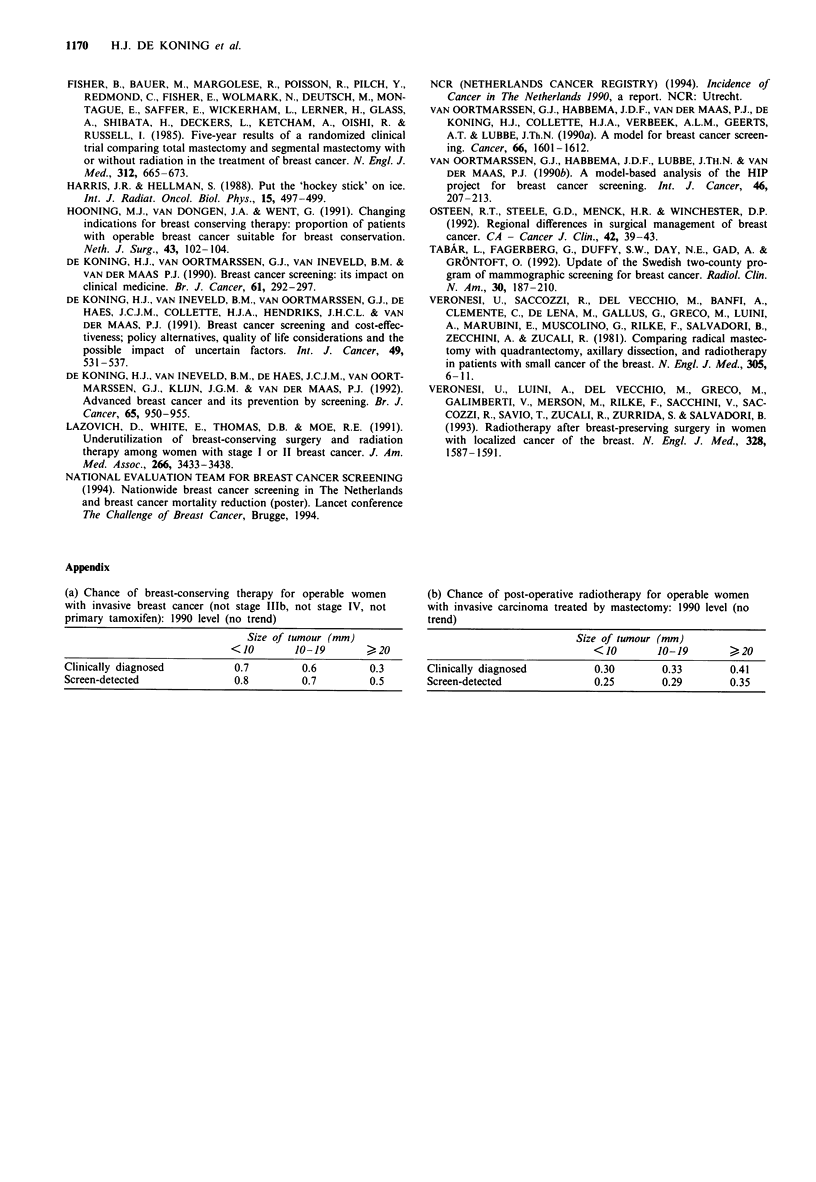


## References

[OCR_00746] Bartelink H., Borger J. H., van Dongen J. A., Peterse J. L. (1988). The impact of tumor size and histology on local control after breast-conserving therapy.. Radiother Oncol.

[OCR_00752] Beemsterboer P. M., de Koning H. J., Warmerdam P. G., Boer R., Swart E., Dierks M. L., Robra B. P. (1994). Prediction of the effects and costs of breast-cancer screening in Germany.. Int J Cancer.

[OCR_00758] Boekema A. G., Dornseiffen G., Mulder H. J., de Vos R. A., Kluft-de Haas B. A. (1992). De eerste ervaring met mammascreening in de regio Enschede. I. Effectiviteit.. Ned Tijdschr Geneeskd.

[OCR_00769] Carter R., Glasziou P., van Oortmarssen G., de Koning H., Stevenson C., Salkeld G., Boer R. (1993). Cost-effectiveness of mammographic screening in Australia.. Aust J Public Health.

[OCR_00775] Chouillet A. M., Bell C. M., Hiscox J. G. (1994). Management of breast cancer in southeast England.. BMJ.

[OCR_00790] Edland R. W. (1988). Does adjuvant radiotherapy have a role in the postmastectomy management of patients with operable breast cancer--revisited.. Int J Radiat Oncol Biol Phys.

[OCR_00796] Farrow D. C., Hunt W. C., Samet J. M. (1992). Geographic variation in the treatment of localized breast cancer.. N Engl J Med.

[OCR_00806] Fisher B., Bauer M., Margolese R., Poisson R., Pilch Y., Redmond C., Fisher E., Wolmark N., Deutsch M., Montague E. (1985). Five-year results of a randomized clinical trial comparing total mastectomy and segmental mastectomy with or without radiation in the treatment of breast cancer.. N Engl J Med.

[OCR_00813] Harris J. R., Hellman S. (1988). Put the "hockey stick" on ice.. Int J Radiat Oncol Biol Phys.

[OCR_00817] Hooning M. J., van Dongen J. A., Went G. (1991). Changing indications for breast conserving therapy: proportion of patients with operable breast cancer suitable for breast conservation.. Neth J Surg.

[OCR_00842] Lazovich D. A., White E., Thomas D. B., Moe R. E. (1991). Underutilization of breast-conserving surgery and radiation therapy among women with stage I or II breast cancer.. JAMA.

[OCR_00870] Osteen R. T., Steele G. D., Menck H. R., Winchester D. P. (1992). Regional differences in surgical management of breast cancer.. CA Cancer J Clin.

[OCR_00875] Tabàr L., Fagerberg G., Duffy S. W., Day N. E., Gad A., Gröntoft O. (1992). Update of the Swedish two-county program of mammographic screening for breast cancer.. Radiol Clin North Am.

[OCR_00893] Veronesi U., Luini A., Del Vecchio M., Greco M., Galimberti V., Merson M., Rilke F., Sacchini V., Saccozzi R., Savio T. (1993). Radiotherapy after breast-preserving surgery in women with localized cancer of the breast.. N Engl J Med.

[OCR_00881] Veronesi U., Saccozzi R., Del Vecchio M., Banfi A., Clemente C., De Lena M., Gallus G., Greco M., Luini A., Marubini E. (1981). Comparing radical mastectomy with quadrantectomy, axillary dissection, and radiotherapy in patients with small cancers of the breast.. N Engl J Med.

[OCR_00838] de Koning H. J., van Ineveld B. M., de Haes J. C., van Oortmarssen G. J., Klijn J. G., van der Maas P. J. (1992). Advanced breast cancer and its prevention by screening.. Br J Cancer.

[OCR_00828] de Koning H. J., van Ineveld B. M., van Oortmarssen G. J., de Haes J. C., Collette H. J., Hendriks J. H., van der Maas P. J. (1991). Breast cancer screening and cost-effectiveness; policy alternatives, quality of life considerations and the possible impact of uncertain factors.. Int J Cancer.

[OCR_00823] de Koning H. J., van Oortmarssen G. J., van Ineveld B. M., van der Maas P. J. (1990). Breast cancer screening: its impact on clinical medicine.. Br J Cancer.

[OCR_00780] van Dongen J. A., Bartelink H., Fentiman I. S., Lerut T., Mignolet F., Olthuis G., van der Schueren E., Sylvester R., Tong D., Winter J. (1992). Factors influencing local relapse and survival and results of salvage treatment after breast-conserving therapy in operable breast cancer: EORTC trial 10801, breast conservation compared with mastectomy in TNM stage I and II breast cancer.. Eur J Cancer.

[OCR_00864] van Oortmarssen G. J., Habbema J. D., Lubbe J. T., van der Maas P. J. (1990). A model-based analysis of the HIP project for breast cancer screening.. Int J Cancer.

[OCR_00858] van Oortmarssen G. J., Habbema J. D., van der Maas P. J., de Koning H. J., Collette H. J., Verbeek A. L., Geerts A. T., Lubbe K. T. (1990). A model for breast cancer screening.. Cancer.

